# New Approaches in the Classification and Prognosis of Sign Clusters on Pulmonary CT Images in Patients With Multidrug-Resistant Tuberculosis

**DOI:** 10.3389/fmicb.2021.714617

**Published:** 2021-10-04

**Authors:** Qisheng Song, Xiaohong Guo, Liling Zhang, Lianjun Yang, Xiwei Lu

**Affiliations:** ^1^Department of Internal Medicine, Dalian Public Health Clinical Center, Dalian, China; ^2^Department of Internal Medicine, Liupanshui Third Hospital, Liupanshui, China

**Keywords:** sign clusters, CT image, multidrug-resistant tuberculosis, principal component analysis, receiver operating characteristic

## Abstract

**Background:** To date, radiographic sign clusters of multidrug-resistant pulmonary tuberculosis (MDR-TB) patients have not been reported. We conducted a study to investigate the classification and prognosis of sign clusters in pulmonary Computed Tomography (CT) images from patients with MDR-TB for the first time by using principal component analysis (PCA).

**Methods:** The clinical data and pulmonary CT findings of 108 patients with MDR-TB in the Liupanshui Third Hospital were collected (from January 2018 to December 2020). PCA was used to analyze the sign clusters on pulmonary CT, and receiver operating characteristic (ROC) analysis was used to analyze the predictive value of the treatment outcome of MDR-TB patients.

**Results:** Six cluster signs of MDR-TB were determined by PCA: nodules, infiltration, consolidation, cavities, destroyed lung and non-active lesions. Nine months after treatment, the area under the ROC curve (AUC) of MDR-TB patients with a cavity sign cluster was 0.818 (95% CI: 0.733–0.886), and the positive predictive value (PPV) and negative predictive value (NPV) of the treatment outcome were 79.6% (95% CI: 65.7–89.8%) and 72.9% (95% CI: 59.7–83.6%), respectively.

**Conclusion:** PCA plays an important role in the classification of sign groups on pulmonary CT images of MDR-TB patients, and the sign clusters obtained from PCA are of great significance in predicting the treatment outcome.

## Introduction

Due to its high incidence rate, high recurrence rate, high mortality rate, long course of treatment and high cost, multidrug-resistant pulmonary tuberculosis (MDR-TB, defined as TB strains resistant to at least isoniazid and rifampin) is still one of the main public health problems in tuberculosis prevention and control (Pontali et al., 2019; Harding, 2020; Migliori et al., 2020; Singh et al., 2020). In addition, there are ~1.9 million latent MDR-TB-infected people around the world (Knight et al., 2019); consequently, the diagnosis and treatment of patients with MDR-TB remain daunting challenges. Imaging is of great reference value for the early diagnosis and evaluation of the treatment outcome of MDR-TB patients. At present, there are many studies describing the signs of CT imaging in patients with MDR-TB (Zahirifard et al., 2003; Song et al., 2016; Wang et al., 2018; Mehrian et al., 2020). However, during clinical practice, there is nearly no possibility that only a single sign exists in a CT image of patients with MDR-TB. Typically, a set of signs are present in some sort of imaging, and there are always obvious correlations among them.

In medical research, the same individual often involves multiple random variables, and these variables regularly have a certain correlation. According to the relationship between the original variables, principal component analysis (PCA) can be used to calculate a few principal components and summarize the main information for the original variables. Compared with other statistical methods, PCA neither increases nor reduces the total information, making it one of the most important dimensionality reduction methods (Wold et al., 1987). Therefore, PCA could be used to analyze the sign clusters on pulmonary CT images in MDR-TB cases.

PCA has been used in many medical studies, but the study of PCA in radiographic sign clusters of MDR-TB patients has not been reported (Yamamura et al., 1991; Yamamoto et al., 2010; Dillmann et al., 2014).

In addition, it might be more accurate to analyze the predictive value of treatment outcomes in MDR-TB patients by using the sign clusters obtained from PCA.

This study retrospectively analyzed the clinical and imaging features of 108 patients with MDR-TB (without HIV) from January 2018 to December 2020 by using PCA, and receiver operating characteristic (ROC) analysis was used to evaluate the abovementioned sign clusters for the positive predictive value (PPV) of the treatment outcome of MDR-TB patients and to provide an important theoretical basis for imaging research on patients with MDR-TB.

## Materials and Methods

### Ethics Statement

The Ethics Committee of Liupanshui Third Hospital approved the study, and individual consent for this retrospective analysis was waived because only imaging findings and demographic data were retrieved from the picture archiving and communication system (PACS) server and medical records.

### Clinical Information

**Figure 1:**
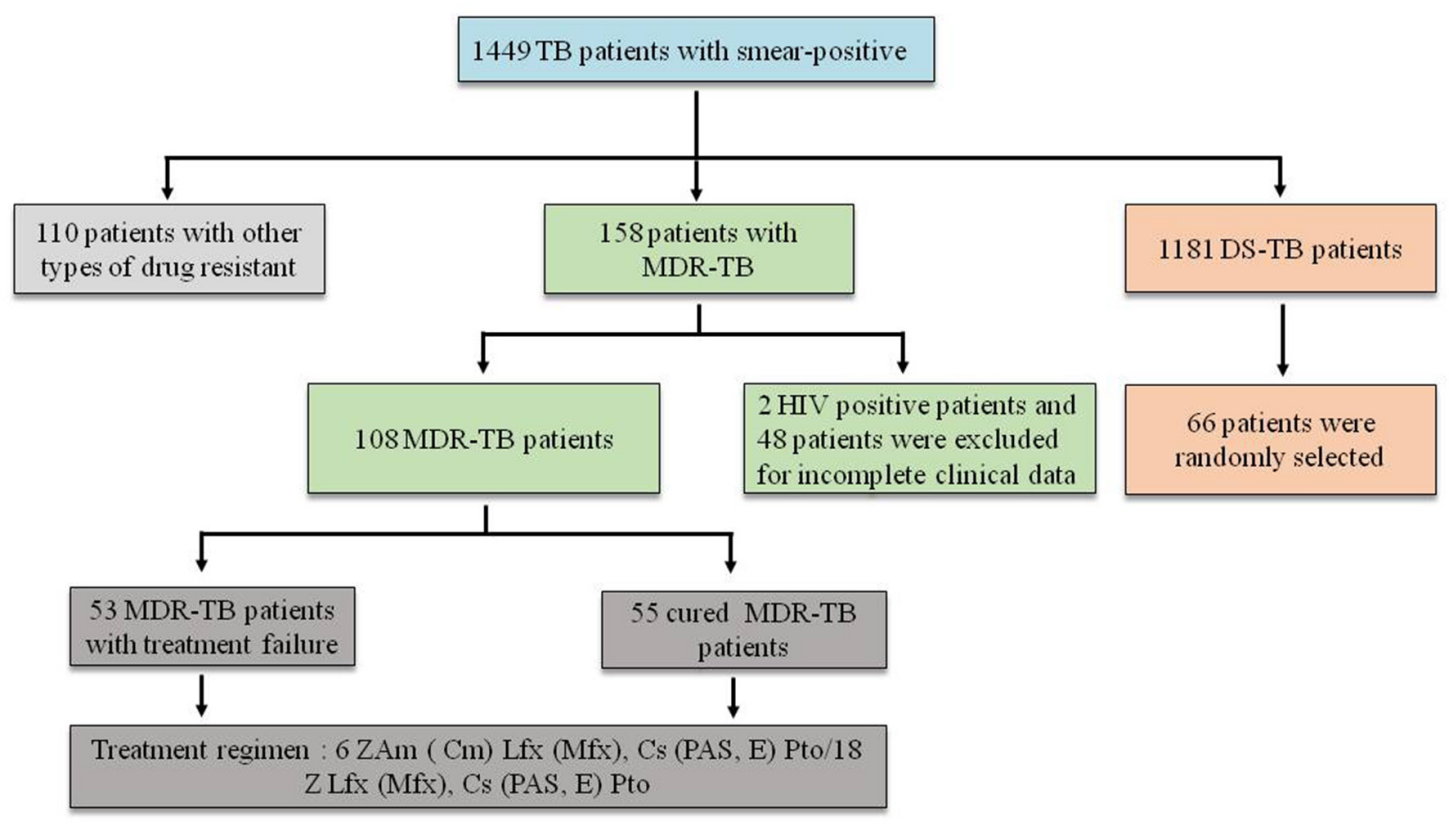
Screening of patients. Z, pyrazinamide; Am, amikacin; Cm, capreomycin; LFX, levofloxacin; MFX, moxifloxacin; CS, cycloserine; PAS, p-aminosalicylic acid; E, ethambutol; PTO, prothionamide.

The research was conducted at the Liupanshui Third Hospital, which is a referral hospital for the management of TB patients and suspects from Liupanshui city, which has a population of more than three million. From January 2018 to December 2020, 1986 patients with a diagnosis of smear-positive pulmonary TB were screened. Of these, 1449 patients with positive sputum cultures were available for review, including 1,181 cases with drug-sensitive TB (DS-TB), 158 cases with MDR-TB and 110 patients with other forms of drug-resistant TB. Of the 156 patients with MDR-TB, 2 HIV-positive cases and 48 cases other cases were excluded due to incomplete clinical data (35 patients had not completed the course of treatment, and 13 patients experienced treatment interruption). The remaining 108 subjects comprised 55 cured patients and 53 patients with treatment failure. Sixty-six patients were randomly selected among the 1,181 patients, with DS-TB as controls. All the subjects in this study were HIV-negative (Figure 1).

The research hospital is located in an economically underdeveloped area of China, so it has not been able to obtain core drugs such as beta-quinoline. The treatment regimen still adopted the guidelines recommended by the World Health Organization (WHO) in 2014 (World Health Organization, 2014) (Figure 1).

### General Information

The clinical data included age, sex, smoking status, alcohol abuse status, and previous TB treatment history.

### CT Image Acquisition

All subjects in this study underwent High-Resolution Computed tomography (HRCT) scans before treatment, in the 3rd, 6th, 9th, and 12th months and post-treatment. Imaging assessments were evaluated for the following signs: (1) airway damage, including as thickening of the airway wall, stenosis or expansion of the lumen and thickening of the interstitium around the tracheal vascular bundle; (2) small nodules <1 cm in diameter, including trees in buds, branch line nodes, nodules with poorly defined margins, cluster nodules, reticulonodular opacity (combined reticular and nodular opacity) and miliary nodules; (3) large nodules between 1 and 3 cm in diameter; (4) patchy opacities, including patchy shadows and irregular flaky infiltration; (5) ground glass opacities (GGOs); (6) pure consolidations, including focal consolidation, segment consolidation, and lobe consolidation; (7) consolidations with cavities: consolidations with single or multiple cavities (including moth-eaten cavities); (8) cavitary lesions <3 in number; (9) multiple cavities ≥ 3 in number but not including consolidations or destroyed lung; (10) pleural involvement (including pleural thickening or pleural effusion); (11) fibrotic lesions, including fibrotic nodules with clear boundaries, fiber chords, fibrous plaque, and fibrous masses; (12) bronchiectasis not included in consolidations or destroyed lung; (13) air trapping; and (14) destroyed lung.

### Scoring Methods

Because the incidence rate of a single sign could not reflect the scope and severity of the lesion, we compared various signs according to the six-zone scoring methods proposed by Casarini et al. (1999). On the HRCT scans, the lungs were divided into six zones (namely, the upper, middle, and lower zones of the right and left lungs). These areas of the lungs were defined as the “upper zones” above the level of the carina, the “middle zones” between the level of the carina and the level of the inferior pulmonary veins, and the “lower zones” below the level of the inferior pulmonary veins. The HRCT score was determined by visually estimating the extent of disease in each zone. The HRCT scans were analyzed by three independent chest radiologists on the premise of unknown disease types, and final conclusions on the findings were reached by consensus. The arbitrary score was based on the percentage of lung parenchyma that showed evidence of each recorded abnormality: involvement of (1) <25%, (2) 25–50%, (3) 50–75%, and (4) more than 75% of the image. A total score for each area was generated by adding the partial scores of each sign mentioned above, theoretically ranging between 0 and 4. Altogether, the total score for each lung ranged between 0 and 24. For example, “X” indicated that the distribution range was more than 75% in six zones of both the right lung and left lung, and the total score was 24 (4 × 6).

### Assessment of the Treatment Results

Cures and treatment failure were determined according to WHO guidelines in 2018 (World Health Organization, 2018). A cure occurs when a patient who has completed treatment exhibits no evidence of failure and has three or more consecutive negative cultures taken at least 30 days apart after the intensive phase. A failure occurs when treatment is terminated or when there is a need for a permanent regimen change of at least two anti-TB drugs because of a lack of conversion by the end of the intensive phase, bacteriological reversion in the continuation phase after conversion to negative, evidence of additional acquired resistance to fluoroquinolones or second-line injectable drugs, or adverse drug reactions.

### Statistical Methods

SPSS 22.0 and MedCalc software were used for data analysis. Measurement data for variables with normal distributions were compared using a *t*-test, and those without normal distributions were compared using a non-rank-sum parameter test. Count data were compared using a chi-squared test. PCA was used to analyze the sign clusters, and the predictive value of the treatment outcome was analyzed by ROC analysis. Statistical significance was set at two-tailed *P* < 0.05.

## Results

### Comparison of the Clinical Characteristics of the Patients With DS-TB and MDR-TB

As the age of the patients did not conform to a normal distribution (*P* < 0.05), the non-rank-sum parameter test was used for comparison, and there was no significant difference between the DS-TB and MDR-TB groups (*P* > 0.05). Moreover, there was no significant difference between the DS-TB and MDR-TB groups (*P* > 0.05) with respect to the other items, except that the treatment history was more frequent in the MDR-TB group than in the control group (*P* < 0.05) after the chi-squared test (Table 1).

**Table 1:** Comparison of clinical characteristics of the patients with DS-TB and MDR-TB.

**Patient characteristics**	**DS-TB (*n* = 66)**	**MDR-TB (*n* = 108)**	***P*-value**
Gender (male) %	46 (69.7)	76 (70.4)	0.925
Age (years, mean ± SD)	40.909 ± 20.412	40.981 ± 15.168	0.658
Smokers vs. non-smokers %	26 (39.4)	43 (39.8)	0.956
Alcohol abuse %	8 (12.1)	9 (8.3)	0.414
Previous treatment history %	6 (9.1)	69 (63.9)	<0.001

*Continuous data are presented as the mean ± SD. Categorical data are presented as numbers (%)*.

*DS-TB, patients with multidrug-resistant tuberculosis; MDR-TB, patients with drug-susceptible tuberculosis*.

### Comparison of Imaging Features in MDR-TB and DS-TB Cases

As all scores of the patients' signs did not conform to a normal distribution (*P* < 0.05), the non-rank-sum parameter test was used for comparison. The results showed that the incidence and score of pure consolidation in DS-TB patients were higher than those in the MDR-TB group (*P* < 0.05); in contrast, the incidence and score of fibrous lesions and bronchiectasis in the MDR-TB group were higher (*P* < 0.05). In multiple cavities, the incidence was higher in the MDR-TB group than in the DS-TB group (*P* < 0.05), but there was no difference in their scores (*P* > 0.05). Other signs, including airway damage, small nodules, large nodules, patchy opacities, GGOs, consolidations with cavitary lesions, air trapping, and destroyed lung, showed no statistically significant differences in incidence or scores between the two groups (*P* > 0.05) (Table 2).

**Table 2:** Comparison of imaging features in MDR-TB and DS-TB cases.

**Signs of imaging**	**Scores of DS-TB (*n* = 66)**	**Scores of MDR-TB (*n* = 108)**	**P1/P2 value**
Airway damage (mean ± SD)/%	2.303 ± 2.083/50 (75.8)	2.815 ± /90 (83.3)	0.459/0.221
Small nodules (mean ± SD)/%	7.030 ± 4.930/66 (100%)	6.389 ± 4.415/107 (99.1)	0.427/0.433
Large nodules (mean ± SD)/%	2.182 ± 1.880/54 (81.8)	2.009 ± 1.753/86 (79.6)	0.511/0.724
Patchy opacities (mean ± SD)/%	0.939 ± 1.424/26 (39.4)	0.639 ± 1.036/40 (37.0)	0.364/0.756
GGOs (mean ± SD)/%	0.773 ± 1.078/26 (39.4)	0.592 ± 1.238/30 (27.8)	0.097/0.112
Pure consolidations (mean ± SD)/%	0.909 ± 1.286/28 (42.4)	0.426 ± 0.949/24 (22.2)	0.003/0.005
Consolidations with cavities (mean ± SD)/%	0.939 ± 1.380/27 (40.9)	1.287 ± 1.919/51 (47.2)	0.338/0.417
Cavity (<3 cavities) (mean ± SD)/%	0.273 ± 0.513/17 (25.8)	0.232 ± 0.466/23 (21.3)	0.632/0.497
Cavity (≥3 cavities) (mean ± SD)/%	0.849 ± 1.542/18 (27.3)	1.167 ± 1.513/47 (43.5)	0.086/0.032
Pleural involvement -/%	-/10 (15.2)	-/20 (18.5)	0.568
Fibrotic lesions (mean ± SD)/%	1.227 ± 1.928/31 (47.0)	2.278 ± 2.254/75 (69.4)	<0.001/0.003
Bronchiectasis (mean ± SD)/%	0.439 ± 1.069/11 (16.7)	1.204 ± 1.656/49 (45.4)	<0.001/ <0.001
Air trapping (mean ± SD)/%	1.197 ± 2.185/22 (33.3)	1.398 ± 2.679/32 (29.6)	0.837/0.608
Destroyed lung (mean ± SD)/%	0.152 ± 1.011/2 (3.0)	0.778 ± 2.592/10 (9.3)	0.108/0.116

*Continuous data are presented as the mean ± SD. Categorical data are presented as numbers (%). DS-TB, patients with multidrug-resistant tuberculosis; MDR-TB, patients with drug-susceptible tuberculosis; P1, p-value used to compare scores of DS-TB and MDR-TB patients; P2, p-value used to compare the incidences of DS-TB and MDR-TB patients; GGOs, ground glass opacities*.

### PCA of Signs in MDR-TB Patients

Using the scores of the foregoing 14 signs of 108 MDR-TB patients before treatment as variables, PCA was performed by using SPSS 22.0 software.

### Statistical Results

The Kaiser-Meyer-Olkin (KMO) test was used to check the partial correlation between variables, and Bartlett's spherical test was used to test the correlation matrix between data. In this study, the KMO value was 0.608 (>0.5), and the chi-squared value of Bartlett's spherical test was 389.651, with *P* = 0.000 (<0.01). Both tests suggested that the sample was suitable for PCA.

### Principal Component Extraction

**Figure 2:**
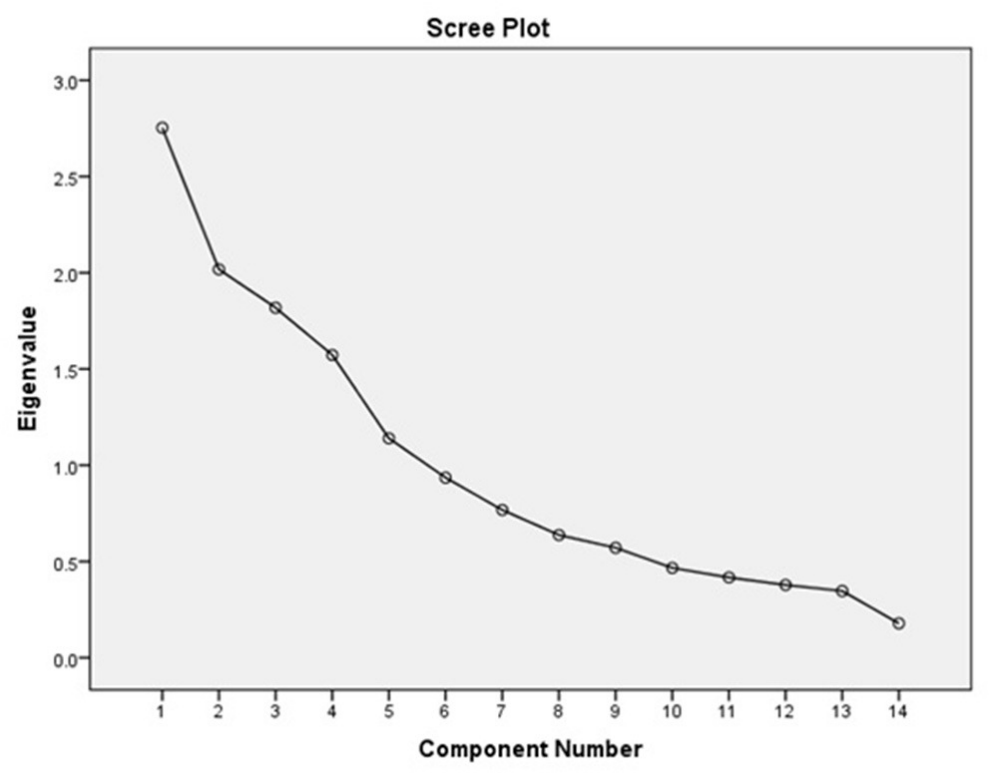
Scree plot.

According to the screen plot (the data after the seventh principal component tended to be flat) and the rule stating that the characteristic root of the principal component be >1, the first six principal components were selected, and the cumulative contribution rate was 73.123%. The specific results are shown in Figure 2 and Table 3.

**Table 3:** Total variance explained.

	**Initial eigenvalues**			**Rotation sums of squared loadings**		
**Component**	**Total**	**% of variance**	**Cumulative %**	**Total**	**% of variance**	**Cumulative %**
1	2.753	19.668	19.668	2.206	15.760	15.760
2	2.018	14.411	34.078	1.905	13.608	29.368
3	1.818	12.988	47.066	1.729	12.352	41.720
4	1.573	11.235	58.301	1.625	11.610	53.330
5	1.140	8.141	66.442	1.500	10.716	64.046
6	0.935	6.681	73.123	1.271	9.077	73.123
7	0.768	5.484	78.607			
8	0.637	4.553	83.160			
9	0.571	4.080	87.240			
10	0.466	3.331	90.572			
11	0.417	2.979	93.551			
12	0.378	2.700	96.250			
13	0.347	2.476	98.726			
14	0.178	1.274	100.000			

*Cumulative %, cumulative contribution rate at which the first six principal components was 73.123%*.

### PCA

The first principal component represented a non-active sign cluster, including fibrotic lesions, bronchiectasis and air trapping. The second principal component represented the destroyed lung sign cluster, including airway damage and pleural involvement. The third principal component represented cavity sign clusters, including cavities and consolidations with cavities. The fourth principal component represented the infiltration sign cluster, including patchy shadows and GGOs. The fifth principal component was the sign cluster of nodules, including various types of nodules. The sixth principal component represented pure consolidations without cavities. The specific results are shown in Table 4 (data with correlation coefficients <0.5 were excluded).

**Table 4:** Rotated component matrix.

**Rotated component matrix^a^**						
**Signs of imaging**	**Component**					
	**1**	**2**	**3**	**4**	**5**	**6**
Bronchiectasis	0.833					
Air trapping	0.717					
Fibrotic lesions	0.678					
Destroyed lung		0.910				
Airway damage		0.818				
Pleural involvement		0.590				
Cavity (≥3 cavities)			0.798			
Cavity (<3 cavities)			−0.763			
Consolidations with cavities			0.516			
GGOs				0.845		
Patchy opacities				0.650		
Large nodules					0.859	
Small nodules					0.609	
Pure consolidations						0.889

*Extraction Method: Principal Component Analysis*.

*Rotation Method: Varimax with Kaiser Normalization*.

a *Rotation converged in 10 iterations*.

*Data with correlation coefficients <0.5 were excluded; GGOs, ground glass opacities*.

### Sign Cluster Prognosis of Patients With MDR-TB

ROC analysis was performed by using the treatment outcomes of MDR-TB patients as the dependent variable and five foregoing active sign cluster scores as covariates. The results showed that the areas under the ROC curves (AUCs) of the sign clusters with infiltration, consolidations and destroyed lung were too low, and those of all treatment stages were <0.7.

The AUCs of the sign clusters of nodules tested before treatment, in the 3rd, 6th, 9th, and 12th months, and post-treatment were 0.669 (95% CI: 0.571–0.756), 0.684 (95% CI: 0.587–0.770), 0.734 (95% CI: 0.641–0.815), 0.764 (95% CI: 0.673–0.841), 0.777 (95% CI: 0.686–0.851), and 0.798 (95% CI: 0.710–0.870), respectively.

The AUCs of the sign clusters of cavities detected before treatment, in the 3rd, 6th, 9th, 12th months, and post-treatment were 0.546 (95% CI: 0.448–0.643), 0.551 (95% CI: 0.452–0.647), 0.702 (95% CI: 0.606–0.786), 0.818 (95% CI: 0.733–0.886), 0.872 (95% CI: 0.794–0.928), 0.883 (95% CI: 0.807–0.937), respectively.

With the extension of the treatment time, the AUC increased gradually, and a significant difference among the different stages appeared after the 3rd month (*P* < 0.05), but no difference was found between the 9th month and post-treatment (*P* > 0.05).

**Figure 3:**
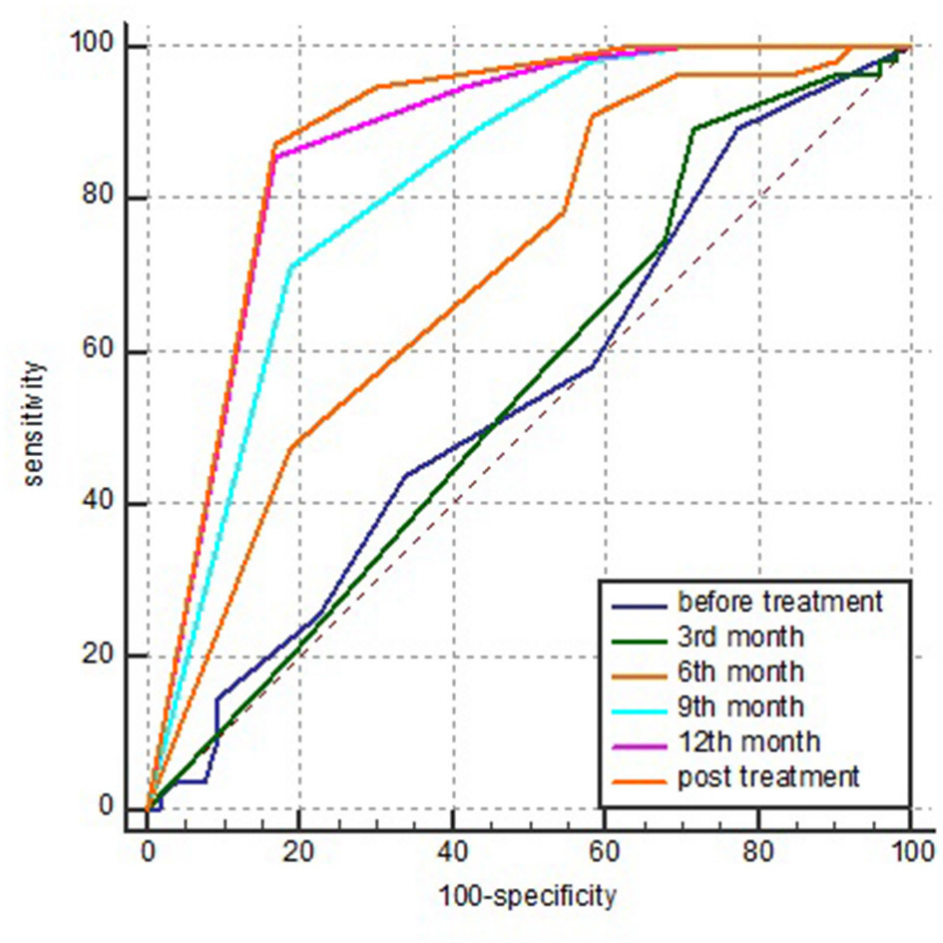
AUC of the sign clusters of cavities detected in various stages of treatment by ROC analysis. AUC, area under the ROC curve and ROC, receiver operating characteristic.

After 6 months of treatment, the sensitivity and specificity were 90.9% (95% CI: 80.0–97.0%) and 41.51% (95% CI: 28.1–55.9%), respectively, and the PPV and negative predictive value (NPV) were 60.3% (95% CI: 48.5–71.2%) and 73.3% (95% CI: 54.1–87.7%), respectively. Nine months after treatment, the sensitivity and specificity were 70.91% (95% CI: 65.7–89.8%) and 72.9% (95% CI: 68.0–90.6%), respectively. The PPV and NPV were 79.6% (95% CI: 65.7–89.8%) and 72.9% (95% CI: 59.7–83.6%), respectively (Figure 3).

## Discussion

Although large studies of MDR-TB patients have been reported, inconsistencies still exist in the clinic due to differences in research areas, objects and methods, especially in imaging.

### Analysis of Clinical Characteristics

In terms of sex, age, smoking status and alcohol abuse status, no difference was found between the two groups, suggesting that these factors might not be risk factors for MDR-TB. As reported in other studies, we found that patients with a previous TB treatment history were significantly more likely to have MDR-TB (Eshetie et al., 2017; Pradipta et al., 2018).

### Features of the CT Signs

#### Consolidations

Unlike other studies of pure consolidations (Yeom et al., 2009; Song et al., 2016), we found that the incidence and scores were relatively high in DS-TB patients (*P* < 0.05) and were similar to those reported by Sulistijawati et al. (2019). Significantly, other studies did not clearly indicate whether the consolidations in their research were accompanied by cavities, and the sign of consolidations in our previous studies included all the consolidations with or without cavities. The difference might stem from the fact that consolidations in other studies were not categorized; on the other hand, as Yeom et al. (2009) put forward, the levels of epidemiology vary across different countries and populations. Therefore, research data from different countries should be provided for more accurate results.

#### Fibrotic Lesions and Bronchiectasis

Similar to other studies, our research showed that both the incidence and scores of fibrotic lesions and bronchiectasis in MDR-TB patients were relatively higher than those in the control group (Chung et al., 2006). Both fibrotic lesions and bronchiectasis were related to collagen deposition and fibrous scarring, which could occur in all stages of anti-TB treatment (Hunter, 2011; DiFazio et al., 2016). Understandably, most MDR-TB patients have a history of anti-TB and treatment with longer courses and experience a lengthy repair process in the lungs; these fibrotic changes are also important reasons for the sequelae of cured MDR-TB patients.

#### Multiple Cavities

Consistent with most literature studies (Chung et al., 2006; Yeom et al., 2009; Wang et al., 2018; Sulistijawati et al., 2019), our study showed that more cavities were found in patients with MDR-TB than in DS-TB patients (*P* < 0.05), although statistical analysis showed no difference in their scores (*P* > 0.05). A possible reason was that the number of patients in the control group was relatively low.

#### Analysis of Sign Clusters Obtained From PCA

Despite many descriptions of the signs of pulmonary tuberculosis (Hatipoglu et al., 1996; Jeon et al., 2016; Meghji et al., 2016; Restrepo et al., 2016), nearly no patients with pulmonary tuberculosis presented with only a single sign. The majority of patients were characterized as having a sign cluster manifesting as one certain main sign accompanied by a variety of other signs, and the pathological bases of some signs were similar or even identical (Hunter, 2011, 2020); all cases involved proliferation (granulomas), exudation, necrosis or a mixed process of mutual transformation. Due to the descriptive signs and comparison of the studies that did not conform, the results were inconsistent.

Therefore, on the one hand, we hope that a unified sign or sign clusters could be applied in imaging studies of MDR-TB patients to compare the results of multiple studies. On the other hand, we would like to use fewer representative signs or sign clusters to simplify the imaging research process.

As an important statistical method of data dimension reduction, PCA could explore the basic structure of observation data by studying the internal dependence of many variables and determine a few hypothetical variables to represent basic data.

In our study, we obtained six CT sign clusters in MDR-TB patients. By using PCA, five active sign clusters (nodules, cavities, infiltration opacities, pure consolidations and destroyed lung) and one non-active sign cluster. To the best of our knowledge, this is the first study to use PCA to classify the signs of MDR-TB patients worldwide. Compared with signs reported in other studies, this classification can simplify the analysis of signs in patients with MDR-TB, which is of great significance for imaging studies of MDR-TB patients.

#### Consolidation With Cavities

This sign included both consolidations and cavities that could not be classified as pure consolidations or simple cavities in terms of imaging. In a pathology study, Hunter (2011) speculated that consolidations, especially consolidations with cavities, were actually caused by bronchial tuberculosis, which leads to airway obstruction and then causes fatty and caseous necrotizing pneumonia at different stages, including various pathological components: fatty components, caseous tissue, cavities, caseous granulomas and fibrotic tissue. This complex pathological component makes it impossible for us to classify it accurately in imaging. However, it was found that consolidations with cavities were significantly correlated with cavities by our PCA, which is consistent with the “Instructions to Panel Physicians for Completing New U.S. Department of State Medical Examination for Immigrant or Refugee Applicants” (Centers for Disease Control and Prevention, 2006). Therefore, this result provides an important basis for the classification of imaging signs in MDR-TB patients.

#### Analysis of Predictive Values

Chen et al. used positron emission tomography (PET) CT and HRCT to predict the treatment effect of 28 MDR-TB patients in 2017 (Chen et al., 2014), which showed very good predicted results for treatment at the 2nd month and 6th month, respectively. However, PET CT is not suitable for the clinic because of its high price and radiation.

In their study, the AUC was 0.82 (95% CI: 0.58–1.0) at the 6th month when the CT reading score was used to predict the outcome of treatment by ROC analysis. However, our study suggested that the AUCs of treatment based on the signs of cavities at the 6th and 9th months were 0.702 (95% CI: 0.606–0.786) and 0.818 (95% CI: 0.733–0.886), respectively. Obviously, the AUC at the 6th month in our study was significantly lower than the corresponding value obtained by Chen et al., but the areas at the 9th month were similar. One reason for these diversities was that our evaluation methods were different: the signs described by Chen et al. were six single signs, and they did not indicate whether consolidations with cavitation were classified as consolidations or cavities. However, the objects we evaluated were five activity sign clusters obtained by PCA. On the other hand, the most important reason might be the different therapeutic regimens. Restricted by economic conditions and other factors, the local tuberculosis hospital still applied the treatment regimen recommended by the WHO in 2014 (World Health Organization, 2014). If the subjects in this study were treated by the treatment regimen guided by the WHO in 2020 (Mirzayev et al., 2020), better predictive value could be obtained.

Moreover, we must recognize that although CT has predictive value for the treatment outcome of MDR-TB patients, it is not ideal. We still need to combine sputum culture at present or find new biomarkers in the future to obtain better predictions.

### Limitations

Our study has several important limitations. First, the number of cases was relatively small. Second, the residential areas in which our study subjects lived were economically impoverished. Due to the limitations of low education and poor income levels, most patients could not seek medical advice at the first appearance of symptoms; some patients, especially those who were elderly, even gave up the diagnosis and treatment, which inevitably led to the bias of investigated object selection. Third, 69 (63.9%) MDR-TB patients had a treatment history, and a longer history was associated with more lesion extents and more damage to the lungs, which had some influence on the imaging findings.

However, from another perspective, delayed diagnosis and treatment could lead to more complex signs. For the patients with MDR-TB who had a treatment history, most of the sensitive mycobacterium had been killed, and the image produced by the action of the sensitive mycobacterium should have been resolved. Therefore, we believe that these imaging data might be more meaningful for the extraction of sign clusters. Naturally, research on more patients and multicenter research should be conducted in the future to confirm our results.

Furthermore, different treatment regimens had a significant influence on the predictive value of the treatment outcome. Therefore, the impact of the latest anti-MDR-TB regimen recommended by the WHO in 2020 on the prognosis of imaging in MDR-TB patients needs to be further studied (Mirzayev et al., 2020).

## Conclusion

PCA plays an important role in the classification of sign groups on pulmonary CT images of MDR-TB patients, and the sign clusters obtained from PCA are of great significance in predicting the treatment outcome.

## Data Availability Statement

The original contributions presented in the study are included in the article/supplementary material, further inquiries can be directed to the corresponding author/s.

## Ethics Statement

The studies involving human participants were reviewed and approved by the Ethics Committee of Liupanshui Third Hospital. Written informed consent from the participants' legal guardian/next of kin was not required to participate in this study in accordance with the national legislation and the institutional requirements.

## Author Contributions

QS and XL conceived and designed the study and drafted the manuscript. QS, XG, LZ, and LY collated and collected data. QS did the statistical analysis. All authors played a significant role in data collection and analysis.

## Funding

This study was supported by a grant to the Guizhou Province Science and Technology Plan Project: [2020] No. 4y151.

## Conflict of Interest

The authors declare that the research was conducted in the absence of any commercial or financial relationships that could be construed as a potential conflict of interest.

## Publisher's Note

All claims expressed in this article are solely those of the authors and do not necessarily represent those of their affiliated organizations, or those of the publisher, the editors and the reviewers. Any product that may be evaluated in this article, or claim that may be made by its manufacturer, is not guaranteed or endorsed by the publisher.
